# Decelerated neurodegeneration after intravitreal injection of α-synuclein antibodies in a glaucoma animal model

**DOI:** 10.1038/s41598-017-06702-1

**Published:** 2017-07-24

**Authors:** J. Teister, F. Anders, S. Beck, S. Funke, H. von Pein, V. Prokosch, N. Pfeiffer, F. Grus

**Affiliations:** 1 Experimental Ophthalmology, Department of Ophthalmology, University Medical Center of the Johannes Gutenberg University, 55131 Mainz, Germany; 2 Institute of Neuropathology, University Medical Center of the Johannes Gutenberg University, 55131 Mainz, Germany

## Abstract

Although elevated intraocular pressure (IOP) remains the major risk factor in glaucoma, neurodegenerative processes continue despite effective IOP lowering. Altered α-synuclein antibody (Abs) levels have been reported to play a crucial role. This study aimed at identifying whether α-synuclein Abs are capable to decelerate neuronal decay while providing insights into proteomic changes. Four groups of Sprague Dawley rats received episcleral vein occlusion: (1) CTRL, no intravitreal injection, n = 6, (2) CTRL IgG, intravitreal injection of unspecific IgG, n = 5, (3) Buffer, intravitreal injection of buffer, n = 6, (4), α-synuclein Ab, intravitreal injection of α-synuclein Ab, n = 5. IOP and retinal nerve fiber layer thickness (RNFLT) were monitored and immunohistochemistry, microarray and proteomic analysis were performed. RNFLT was reduced in CTRL, CTRL IgG and Buffer group (all p < 0.01) and α-synuclein Ab group (p = 0.17). Axon and RGC density showed an increased neurodegeneration in CTRL, CTRL IgG and Buffer group (all p < 0.01) and increased neuronal survival in α-synuclein Ab group (p = 0.38 and 0.06, respectively) compared with fellow eyes. Proteomic analysis revealed alterations of cofilin 1 and superoxide dismutase 1 expression. This data indicate that α-synuclein Ab might indirectly modulate the actin cytoskeleton organization and negatively regulate apoptotic processes via cofilin 1 and superoxide dismutase 1.

## Introduction

In glaucoma, a progressive optic neuropathy occurs due to a sequence of neurodegenerative processes finally leading to the slow but irreversible loss of retinal ganglion cells (RGC) and their axons. This neurodegeneration results in impaired vision which might result in blindness if left untreated and will concern around 80 million people by the year 2020^1^. Next to aging and elevated intraocular pressure (IOP) levels as main risk factors, a variety of other factors, encircling race, myopia, thin central corneal thickness and genetic dispositions are involved in the pathomechanism of the disease. Until now, the most effective treatment of glaucoma consists of medical and surgical therapies to lower IOP in order to reduce progressive neurodegeneration^2^. Even after a successful IOP-lowering therapy many patients still show signs of progressive neurodegeneration, indicating again the multifactorial pathogenesis and the complexity of glaucoma (reviewed in ref. 3). Neuroprotective approaches in glaucoma are mainly IOP dependent and involve the lowering and stabilization in order to decelerate neurodegeneration. In recent years, strategies independent of the IOP regulation and focusing on the pathophysiological processes are investigated to retard the progression of glaucoma^4^. Other pathophysiological processes including oxidative stress, glutamate excitotoxicity, abnormal protein accumulations, vascular dysfunctions and abnormal autoantibody (Aab) levels of the autoimmune component trigger apoptosis^5–7^. Especially the role of Aabs has been more and more considered within recent years^8–12^. Under healthy conditions, these Aabs are regulatory factors that are involved in a variety of physiological activities such as homeostasis, transport and modulation of biologically active molecules or immune regulation^13^. However, it has been reported that the equilibrium of the Aab is disturbed in glaucoma, as abnormal Aab levels have been reported in serum, tear fluids, aqueous humor and retinal tissue of glaucomatous patients^14–18^. Candidates of interest are α-synuclein Aabs, which have been found to be downregulated in serum and upregulated in aqueous humor of glaucoma patients^19^. Furthermore, the Aab to α-synuclein is of special interest since elevated levels of the monomeric and oligomeric Abs have been found in serum of patients suffering from Parkinson’s disease five years after onset of the disease^20^. Parkinson’s disease, as well as glaucoma are progressive neurodegenerative disorders including a complex pathomechanism and immune dysregulations. Synucleins represent a large family of cytosolic proteins with α-, β-, and ɣ-synuclein isoforms and are often referred to be involved in the pathogenesis of a variety of neurodegenerative diseases^21–23^. Specifically, α-synuclein is associated with the so called synucleinopathies and is known to constitute Lewy Bodies, specific α-synuclein protein inclusions occurring in Parkinson’s disease. α-synuclein occurs either as monomeric cytosolic protein or as oligomeric protein after exocytotic secretion by neurons^24^. It interacts with astrocytes and microglia, and triggers pro-inflammatory responses^25^. Despite intensive research, the function of α-synuclein protein is not fully understood. α-synuclein knockout mice showed only little abnormalities in neurotransmission, giving a hint for a non-essential role of the α-synuclein protein^26, 27^. Additionally, little is known about the functions of anti- α-synuclein Abs, althought different immunotherapy approaches have been performed in animal models for synucleinopathies^28, 29^. Collectively, these findings suggest that α-synuclein Abs are involved not only in glaucoma and Parkinson’s disease but also in a variety of other neurodegenerative diseases^22^. Purpose of this study was to analyse the impact of exogenous α-synuclein Abs as a potential neuroprotective agent in a glaucoma animal model.

## Results

### Episcleral vein occlusion increased the intraocular pressure

**Figure 1:**
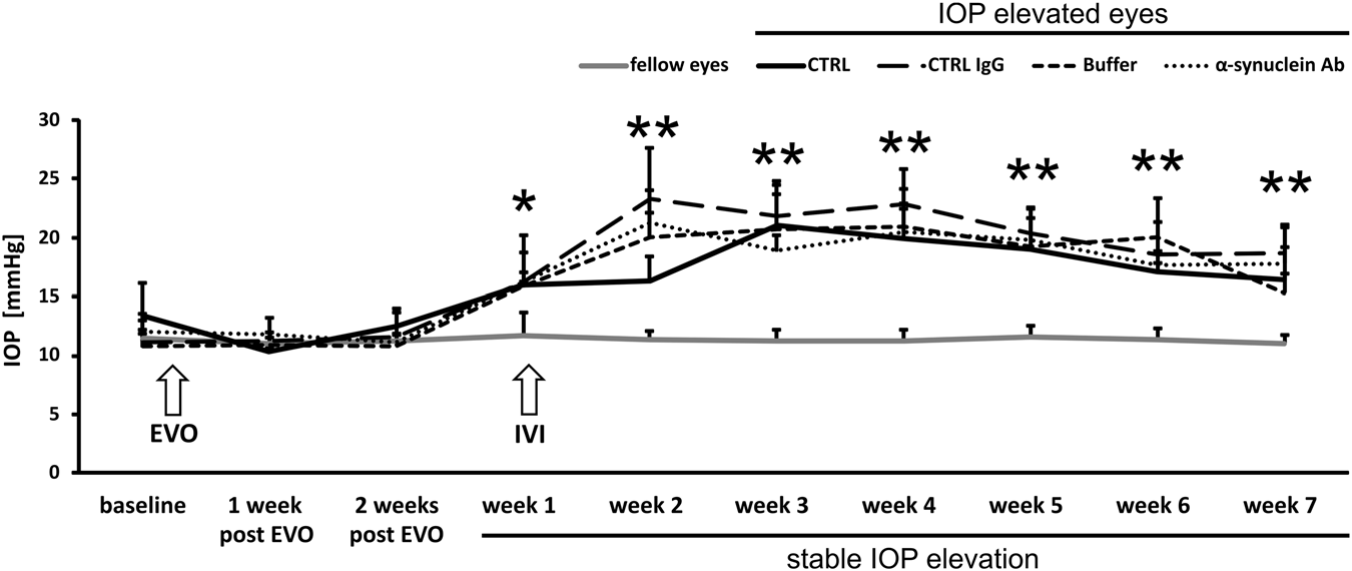
Follow-up of the intraocular pressure before and after episcleral vein occlusion and intravitreal injection. The time course shows the elevation of the IOP after episcleral vein occlusion (EVO). Three weeks after EVO, IOP was significantly elevated and remained high for the next weeks. At the timepoint of stable IOP elevation (week 1), intravitreal injections (IVI) were performed in CTRL IgG, Buffer and α-synuclein Ab group. IOP levels of IOP elevated eyes remained comparable between CTRL (black line), CTRL IgG (broken line), Buffer (hatched line) and α-synuclein Ab (dotted line). The IOP of fellow eyes (grey line) were not affected throughout the study. *Represents p < 0.05, ** represents p < 0.01 compared with fellow eyes at respective time points.

All groups showed comparable baseline levels between 10.8 and 13.4 mmHg. In the first week of stable IOP elevation, levels were significantly elevated in CTRL (15.9 ± 0.3 mmHg, p < 0.01), CTRL IgG (16.2 ± 4.0 mmHg, p < 0.05), Buffer (15.8 ± 3.0 mmHg, p < 0.01) and α-synuclein Ab (16.2 ± 0.9 mmHg, p < 0.01) groups. IOP remained stable throughout the study in CTRL (16.5 ± 2.7 mmHg, p < 0.01), CTRL IgG (18.7 ± 2.2 mmHg, p < 0.01), Buffer (15.3 ± 1.6 mmHg, p < 0.01) and α-synuclein Ab (17.8 ± 3.3 mmHg, p < 0.01) groups. Fellow eyes were not affected by EVO and showed IOP levels in a range from 10.8 ± 0.5 to 11.7 ± 1.5 mmHg (Fig. 1).

### The retinal nerve fibre layer thickness decreased slower in IOP elevated eyes after α-synuclein injection

**Figure 2:**
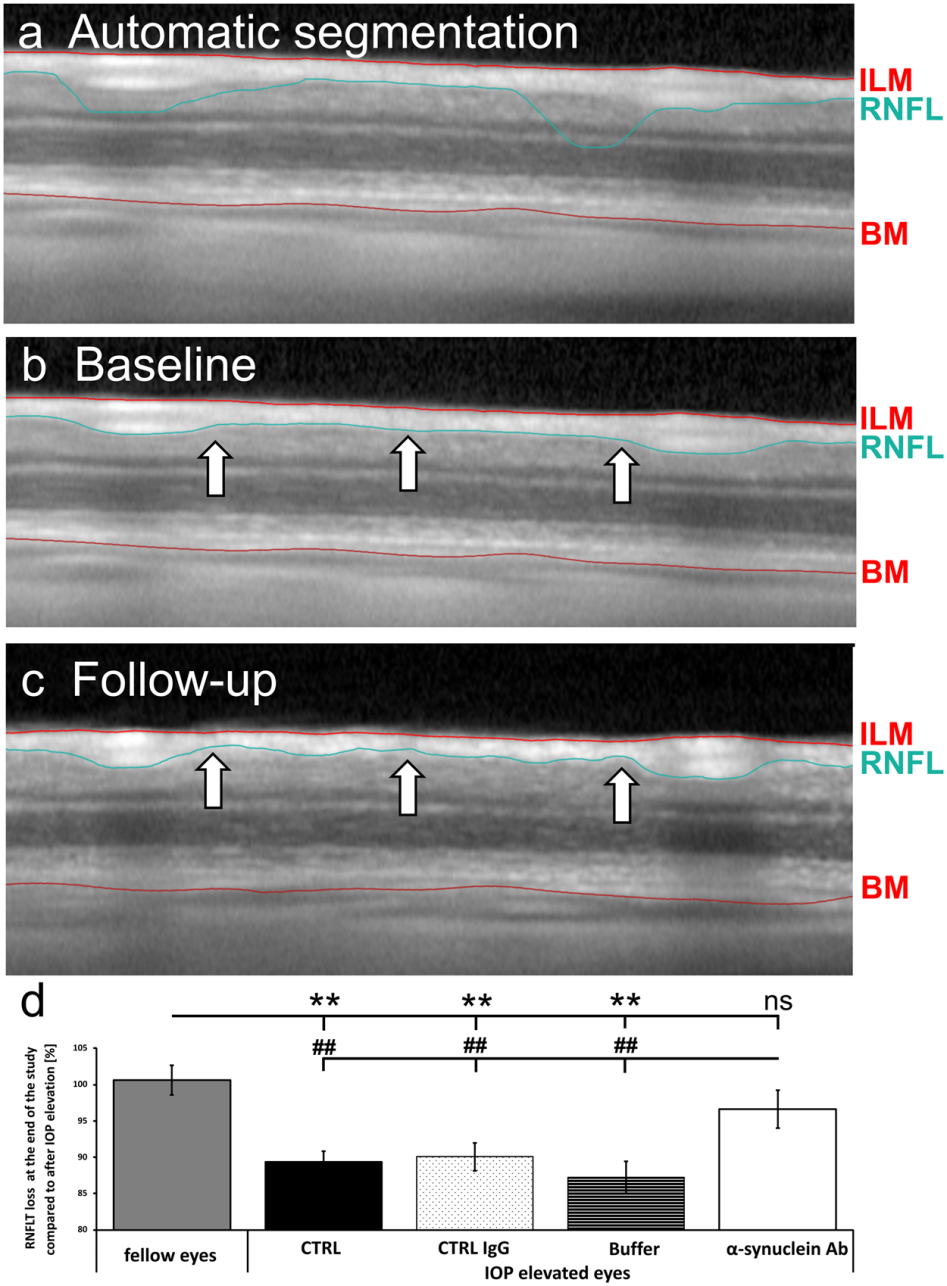
Optical coherence tomography enables circular scans of the retina to detect changes of the retinal nerve fibre thickness. The optical coherence tomography (OCT) enables cross-sectional images of the retina *in vivo* to gather longitudinal information about changes of the retinal nerve fiber layer thickness (RNFLT). RNFLT was defined as the distance between the inner limiting membrane (ILM, red line) and the retinal nerve fiber layer (RNFL, green line). For orientation, the basal membrane (BM, lower red line), indicating the border to the retinal pigment epithelium, is shown. (**a**) Using the Heidelberg Eye Explorer software, the native segmentation algorithm designed to identify specific layers of the human retina, was not always suitable for rodent retina. The RNFL needed manual correction with the circle device. (**b**) The baseline measurement of a CTRL animal. (**c**) The follow-up of the same eye at the end of the study shows distinct changes of the RNFLT as indicated by arrows. (**d**) The loss of RNFLT at the end of the study was compared with after IOP elevation. Fellow eyes were not affected. IOP elevated eyes of CTRL, CTRL IgG and Buffer group showed a significant loss, while damage was less severe in α-synuclein Ab group. With respect to α-synuclein Ab group, RNFLT was significantly higher than in CTRL, CTRL IgG and Buffer groups. **Represents p < 0.01 compared with fellow eyes. ^##^Represents p < 0.01 compared with IOP elevated eyes of α-synuclein Ab group. ns = not significant.

RNFLT did not reveal changes in fellow eyes throughout the study (100.6 ± 2.0%, p = 0.36). RNFL revealed a significantly reduced thickness in IOP elevated eyes of CTRL (89.3 ± 1.5%, p < 0.01), CTRL IgG (90.0 ± 1.9%, p < 0.01) and Buffer group (87.2 ± 2.2%, p < 0.01) compared with fellow eyes, while α-synuclein Ab group was affected less severely (96.6 ± 2.6%, p = 0.17). In fact, IOP elevated eyes of α-synuclein Ab group showed a significantly lower loss of RNFLT than CTRL (p = 0.009), CTRL IgG (p = 0.008) and Buffer groups (p = 0.002, Fig. 2d).

### Axonal loss induced by IOP elevation

**Figure 3:**
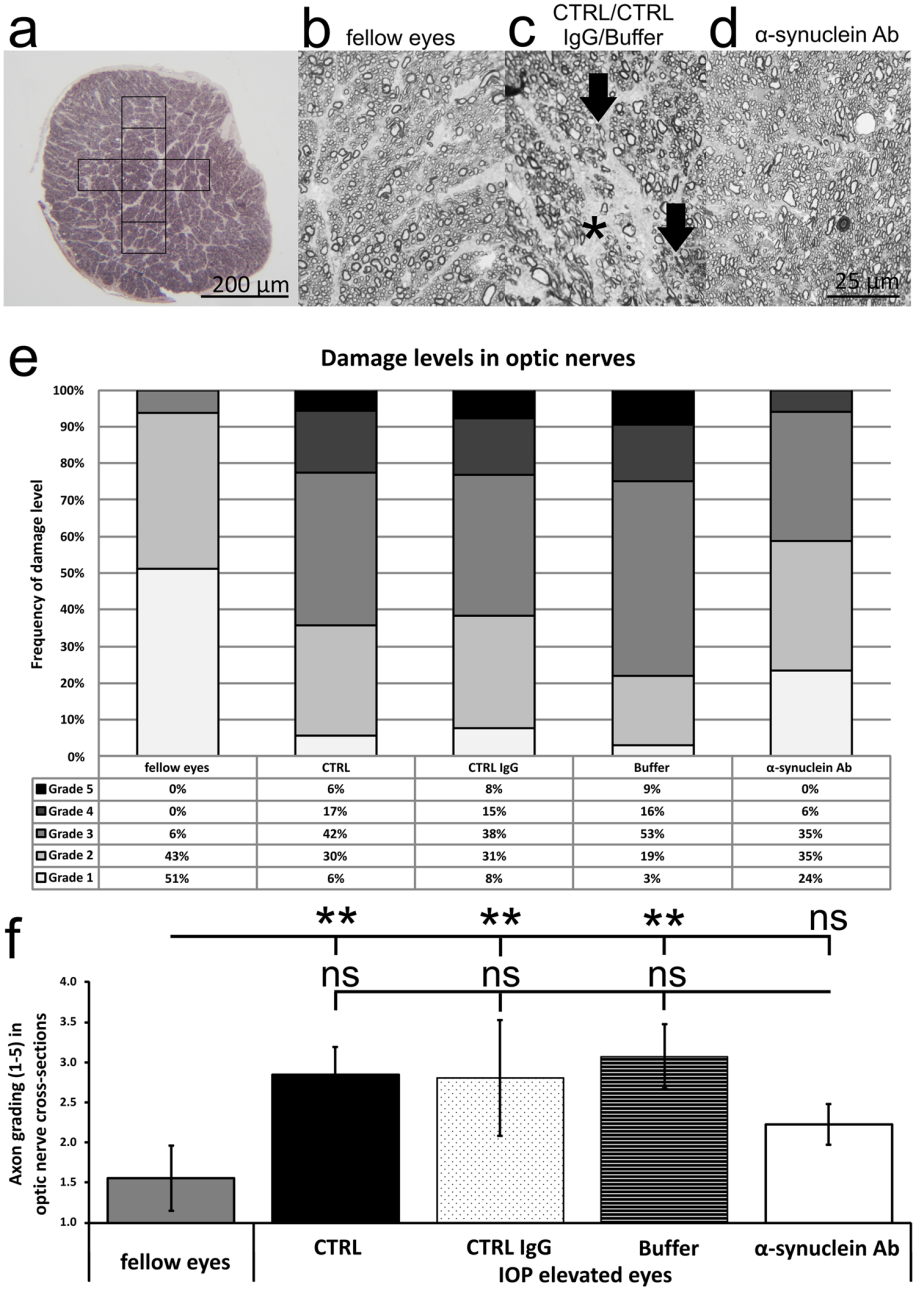
Survival of axon bundles in the optic nerve after IOP elevation. The degree of neurodegeneration was accessed by analysis of the optic nerve axonal damage levelin cross sections. (**a**) A representative low magnification image of an ultrathin optic nerve cross section. Six to seven images were taken for analysis. (**b–d**) Representative high resolution images of axons with different degrees of neurodegeneration, from healthy (fellow eyes, **b**) to severely damaged (CTRL/CTRL IgG/Buffer, arrow indicate axons with collapsed myelin sheaths, star indicates glial scars, **c**). IOP elevated eyes of α-synuclein Ab group showed slightly fewer damage (α-synuclein Ab, **d**). (**e**) Damage levels in optic nerves are arranged according to their damage grade (grade 1: healthy to grade 5: severely damaged). IOP elevated eyes of CTRL, CTRL IgG and Buffer group show the highest degree of neuronal degeneration, while degeneration was reduced in α-synuclein Ab group. (**f**) The statistical analysis revealed significantly elevated damage scores for CTRL, CTRL IgG and Buffer group compared with fellow eyes (indicated as **For p < 0.01). Compared with α-synuclein Ab group, no significant differences could be observed to control groups (ns = not significant). Scale bars as indicated.

The damage level of paraphenylendiamine-stained optic nerve cross sections was analyzed according to a grade 1 (healthy) to grade 5 (severe damage) grading scheme. Healthy optic nerves (grade 1) were found in 51% of fellow eyes, and 6%, 8% and 3% in IOP elevated eyes of CTRL, CTRL IgG and Buffer group, respectively. Thus, 24% grade 1 optic nerves were found for α-synuclein Ab group. Mildly damaged optic neves (grade 3) were found in 6% of fellow eyes, and 42%, 38%, 53% and only 35% in CTRL, CTRL IgG, Buffer and α-synuclein Ab group, respectively. Additionally, severely damaged optic nerves with signs of gliosis (grade 5) were identified in 6%, 8% and 9% of CTRL, CTRL IgG and Buffer group, but not at all in fellow eyes and α-synuclein Ab group (Fig. 3e). Summarizing the damage level in optic nerves, a mean of 1.6 ± 0.4 in fellow eyes was significantly lower than those of CTRL (2.8 ± 0.3, p = 0.0002), CTRL IgG (2.8 ± 0.7, p = 0.009) and Buffer group (3.1 ± 0.4, p = 0.0001). However, α-synuclein Ab group showed a reduced damage level of 2.2 ± 0.3 (p = 0.3). Comparing the damage level of α-synuclein Ab group with the control groups, no significant differences could be observed (p > 0.05, Fig. 3f).

### Retinal ganglion cell loss was highest in peripher parts of the retina and was decelerated in IOP elevated eyes after α-synuclein antibody injection

**Figure 4:**
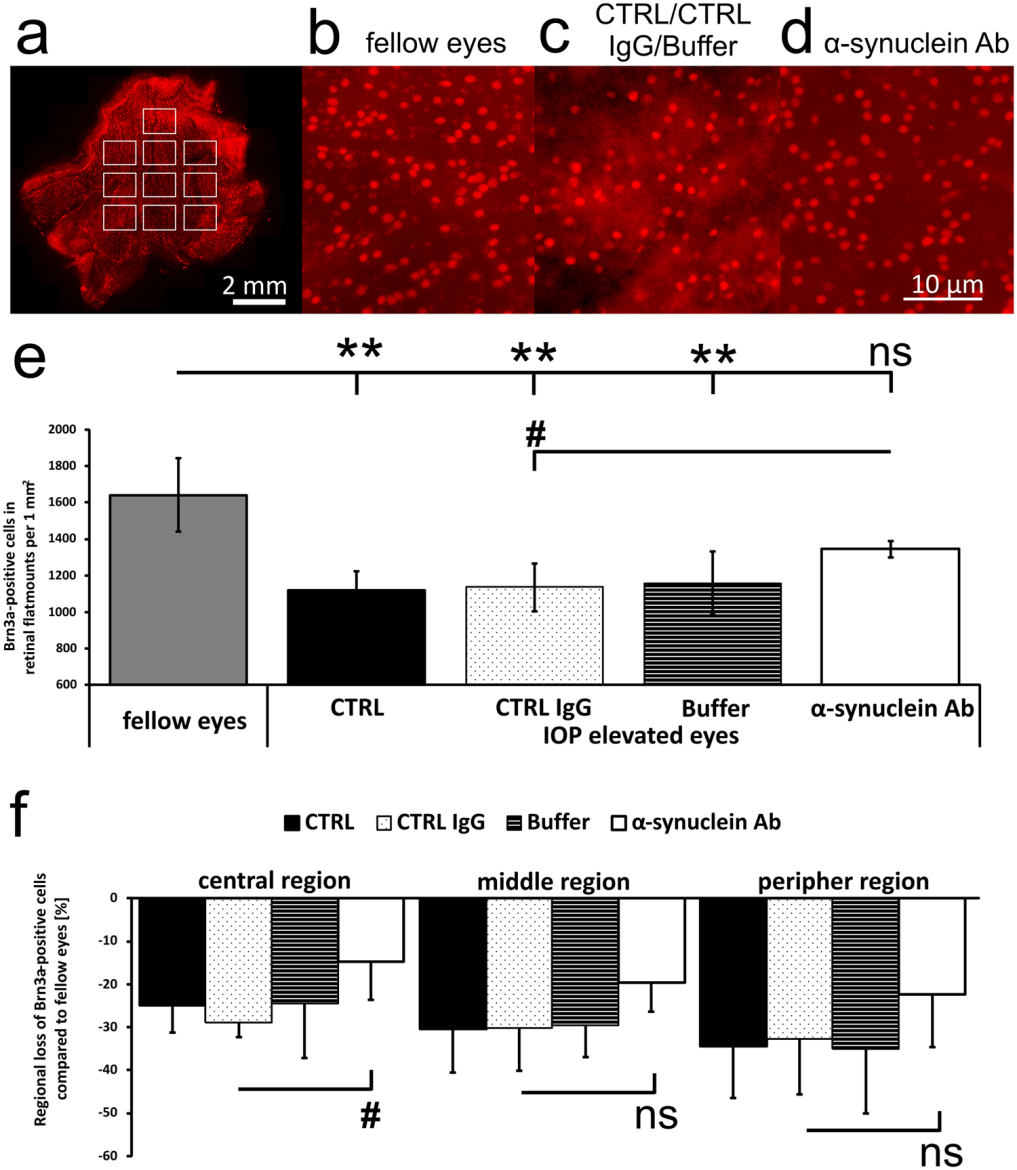
Survival of retinal ganglion cells in the retina after IOP elevation. Neurodegeneration was analyzed using Brn3a-immunostaining as a marker for retinal ganglion cells (RGC) on a quarter of retinal flatmounts. 10 images were taken for analysis and divided into central, middle and peripher position depending on the optic nerve head (ONH). (**a**) A representative stitched image of a quarter of retinal flatmount. Brn3a-staining highlights RGCs in the retina. (**b–d**) Representative high magnification images of Brn3a-positive cells with different densities from healthy (fellow eyes, **b**) to severely damaged (CTRL/CTRL IgG/Buffer group, **c**). IOP elevated eyes of α-synuclein Ab group showed slightly higher numbers of Brn3a-positive cells (α-synuclein Ab, **d**). (**e**) A significant loss of Brn3a-positive cells compared with fellow eyes was observed for CTRL, CTRL IgG and Buffer IOP elevated eyes, but not for α-synuclein Ab IOP elevated eyes. A significantly higher number of Brn3a-positive cells survived in α-synuclein Ab group compared with CTRL IgG group (student’s independent t-test by groups). (**f**) The regional loss in dependency to the optic nerve head was analyzed. Peripher region showed highest loss of Brn3a-positive cells, while the loss decreased gradually towards the optic nerve head. Especially in the central region, α-synuclein Ab showed a significant difference to CTRL IgG group. **Represents p < 0.01 compared with fellow eyes. ^#^Represents p < 0.05 compared with IOP elevated eyes of α-synuclein Ab group. ns = not significant. Scale bars as indicated.

The density of RGC was identified as the numbers of Brn3a-positive cells per 1 mm^2^ in a quarter of a retinal flatmount (Fig. 4a–d). Fellow eyes showed 1642 ± 202 RGCs, while RGC densities of IOP elevated eyes of CTRL (1118 ± 106 RGC, p = 0.0002), CTRL IgG (1135 ± 132 RGC, p = 0.0004) and Buffer (1158 ± 173 RGC, p = 0.0003) group were significantly lower. IOP elevated eyes of α-synuclein Ab group had 1344 ± 45 RGCs (p = 0.06, compared with fellow eyes). Comparing α-synuclein Ab group to CTRL IgG group, a significant differences could be observed (p = 0.01, student’s independent t test by groups, Fig. 4e). Additionally, the loss of RGCs in dependency to their distance to the optic nerve head (ONH) was analyzed. Compared with fellow eyes, central, middle and peripheral position showed a loss of −24.8 ± 6.3, −30.4 ± 10.1 and −34.4 ± 12.0% in CTRL group, −29.0 ± 3.3, −30.2 ± 9.8 and −32.8 ± 12.8% in CTRL IgG group, −24.5 ± 12.7, −29.6 ± 7.4 and −35.1 ± 14.9% in Buffer group and −14.7 ± 9.0, −19.7 ± 6.7 and −22.3 ± 12.4% in α-synuclein Ab group, respectively. Loss of RGCs was significantly lower in central region of CTRL IgG group vs α-synuclein Ab group (p = 0.03), but not for middle and peripher region of the retina (p > 0.05). For all IOP elevated eyes, loss of Brn3a-positive cells was highest in the peripher part of the retina (Fig. 4f).

### Detection of the α-synuclein antibody and the protein in the retina

**Figure 5:**
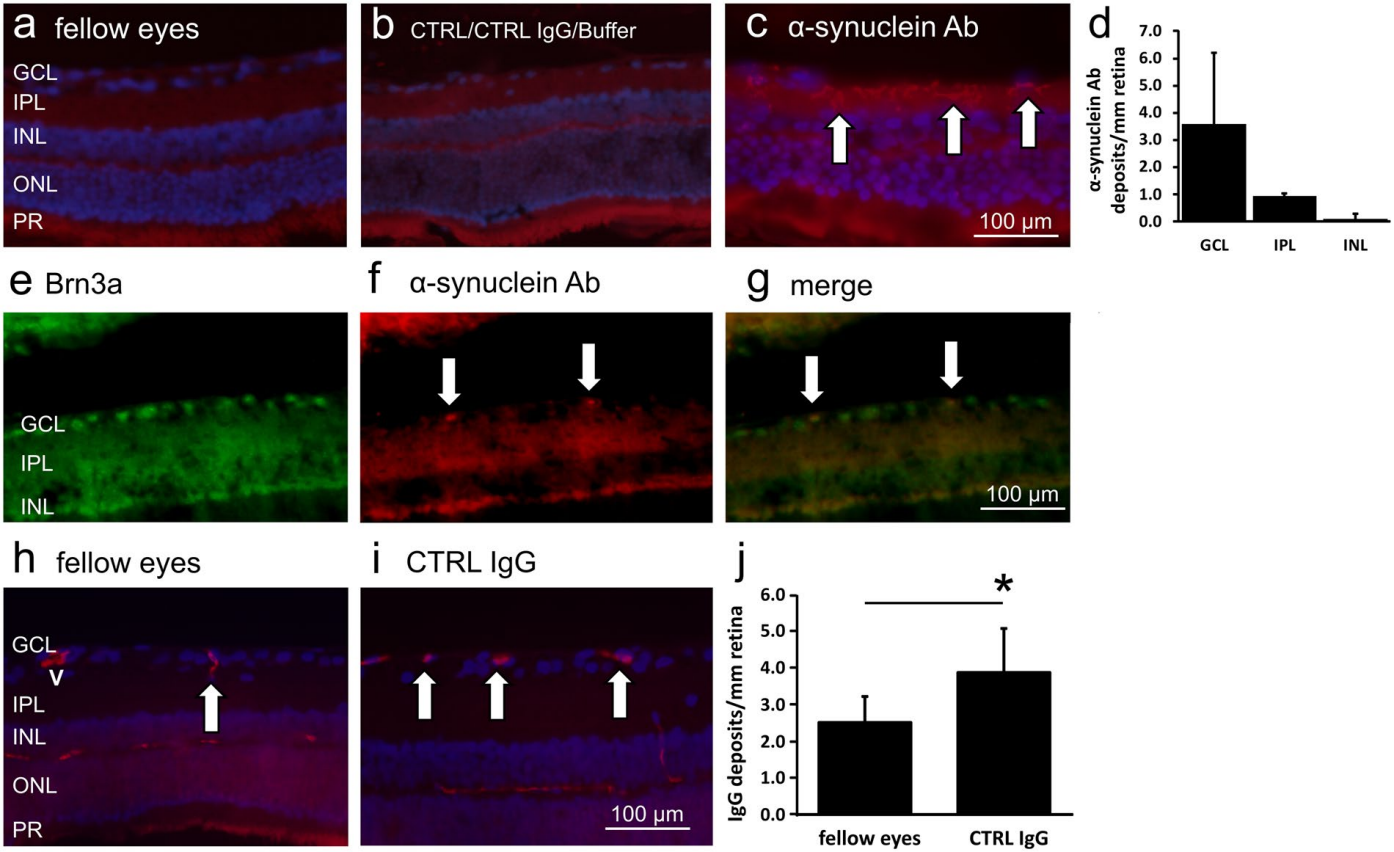
Localization of the α-synuclein antibody in retinal cross sections. The localization of the previously injected α-synuclein Ab was visualized with immunohistochemistry on retinal cross sections using a goat anti-rabbit IgG (H + L) Cy3 secondary Ab. (**a**) The fellow eye revealed weak unspecific background staining. (**b**) CTRL, CTRL IgG and Buffer group showed weak unspecific background staining. (**c**) Specific staining of α-synuclein Ab in the α-synuclein Ab group was observed in the innermost layers of the retina (indicated by arrows). (**d**) Analysis of α-synuclein Ab deposits per mm retinal layer. (**e–g**) A colocalization of Brn3a-positive retinal ganglion cells and α-synuclein Ab could be shown. (**h–j**) IgG deposits were visualized in retinal cross sections of fellow eyes and CTRL IgG-injected eyes (indicated by arrows, a blood vessel is marked with v). Abbreviations: GCL: ganglion cell layer, IPL: inner plexiform layer, INL: inner nuclear layer, ONL: outer nuclear layer, PR: photoreceptors.

Eyes of animals that received the α-synuclein Ab were investigated for remaining deposits of the Ab. Therefore, a staining against the injected primary Ab using a suitable secondary Ab (goat anti-rabbit IgG (H + L) Cy3) was performed in fellow eyes and IOP elevated eyes of all experimental groups. A background signal was detected in photoreceptors, while no α-synuclein Ab deposits were detected in the fellow eyes and control groups (Fig. 5a and b). α-synuclein Ab injected eyes showed the same background signal as fellow eyes and control groups as well as distinct deposits in the inner layers of the retina (Fig. 5c). The analysis of the number of α-synuclein Ab deposits revealed 3.6 ± 2.6 deposits/mm in the ganglion cell layer, 0.9 ± 0.1 depositions/mm in the inner plexiform layer and 0.1 ± 0.2 depositions/mm in both, the inner nuclear layer and the outer nuclear layer (Fig. 5d). No deposits were seen in the outer plexiform layer. A colocalization of Brn3a-positive cells and α-synuclein Ab deposits could be shown (Fig. 5e–g). This data suggests that the α-synuclein Ab bound to cells in the retina was predominantely integrated into the ganglion cell layer and the inner plexiform layer. The number of IgG deposits in the ganglion cell layer were identified in fellow eyes and eyes of CTRL IgG group. Fellow eyes showed 2.5 ± 0.7 IgG deposits/mm retina while CTRL IgG group showed 3.9 ± 1.2 IgG deposits/mm retina, indicating an intracellular uptake of intravitreally injected CTRL IgGs in neuronal cells of the retina (p = 0.02, Fig. 5h–j).

**Figure 6:**
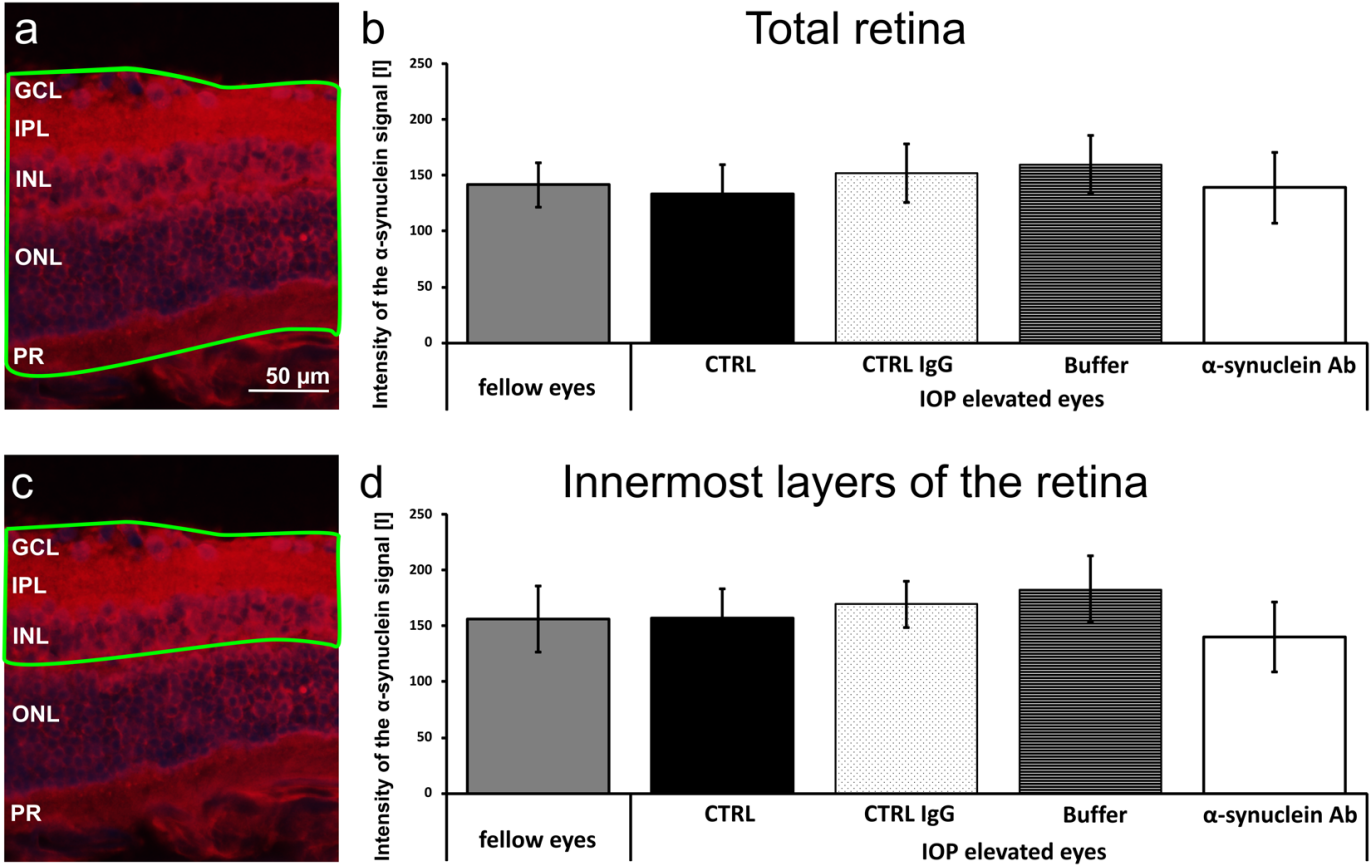
Expression levels of α-synuclein protein in retinal cross sections. The intensity level of α-synuclein protein signal was compared in the total retina after immunohistochemistry on retinal cross sections using rabbit α-synuclein primary Ab and a goat anti-rabbit IgG (H + L) Cy3 secondary Ab. (**a**) The green box indicates the region which is included in the analysis. (**b**) No differences were observed for α-synuclein signal intensity [I] between the experimental groups. (**c**) The innermost layers of the retina includes the GCL, IPL and INL, indicated by the green box. (**d**) No differences were observed for α-synuclein signal intensity between the experimental groups. Abbreviations: GCL: ganglion cell layer, IPL: inner plexiform layer, INL: inner nuclear layer, ONL: outer nuclear layer, PR: photoreceptors.

Furthermore, the α-synuclein protein was visualized using classical immunohistochemistry to investigate expression level changes due to IOP elevation. The intensity of the α-synuclein protein signal in the total retina was comparable in fellow eyes and IOP elevated eyes of CTRL, CTRL IgG, Buffer and α-synuclein Ab injected groups (p < 0.05, Fig. 6a and b). Additionally, no differences of α-synuclein protein signal intensity in the innermost layers of the retina was found between all groups (p < 0.05, Fig. 6c and d).

### Changes of humoral immunoreactivities

**Figure 7:**
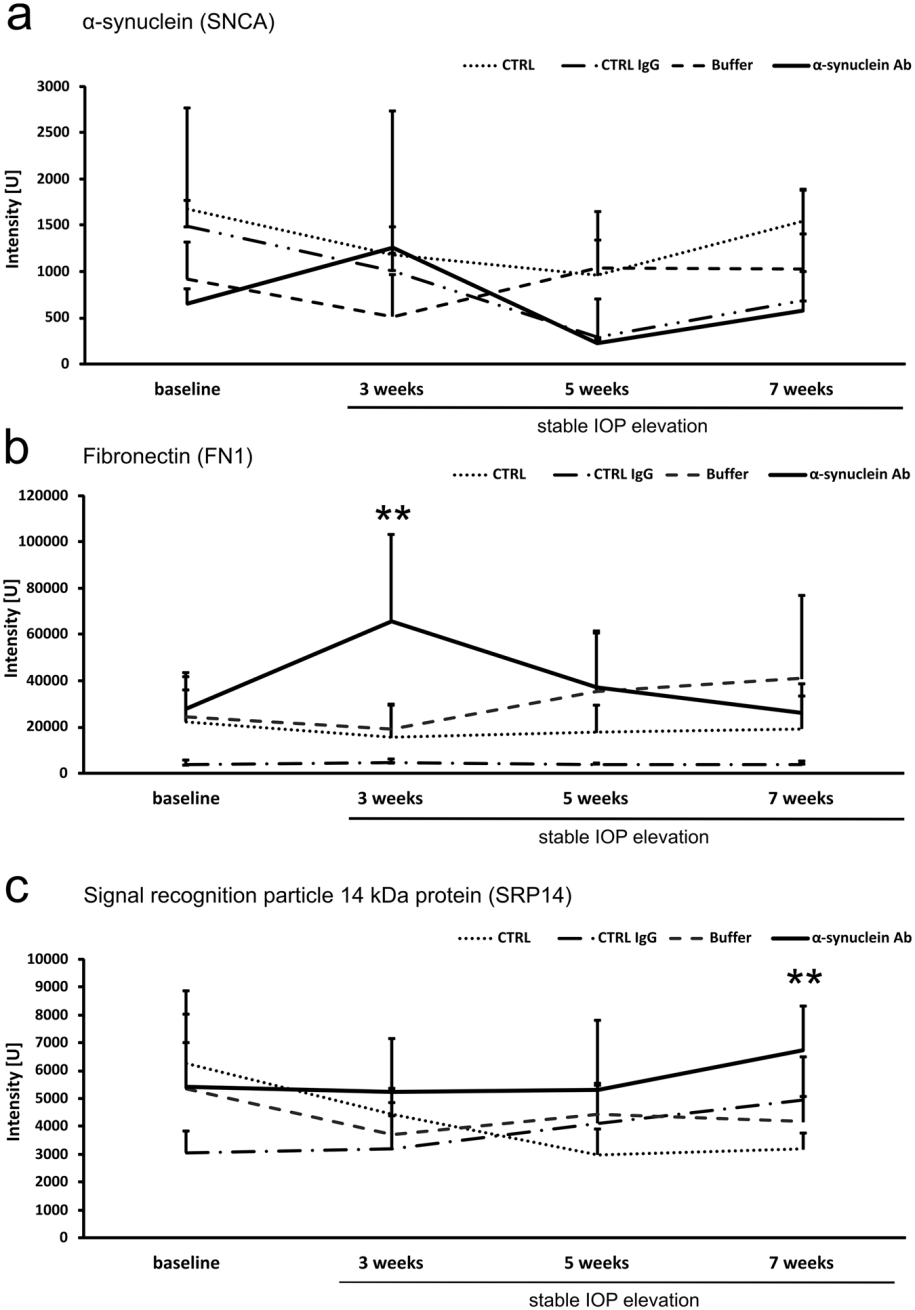
Follow-up of the immunoreactivities of antibodies to α-synuclein, fibronectin and signal recognition particle 14 kDa protein. Quantification of antigen reactivities of control (CTRL,dotted line), CTRL IgG (dash/dotted line), Buffer (hatched line) and α-synuclein injected (α-synuclein Ab, continuous black line) animals at baseline, and 3, 5, and 7 weeks after stable IOP elevation. (**a**) The α-synuclein (SNCA) antigen-Ab immunoreactivities are plotted and show no significant changes for CTRL, CTRL IgG, Buffer and α-synuclein Ab group over time. (**b**) Fibronectin (FN1) is a major surface glycoprotein that has been shown to interact with intracellular actin filament bundels. Compared to all three control groups, α-synuclein Ab group show significantly elevated immunoreactivities three weeks after stable IOP elevation (**, all p < 0.001) with a decrease to baseline levels after 5 and 7 weeks. (**c**) The signal recognition particle 14 kDa protein (SRP14) is plotted. At the end of the study, immunoreactivities of the α-synuclein Ab group were significantly increased compared with CTRL and Buffer group (**p < 0.01).

To identify changes of humoral IgG autoantibody reactivities, an antigen-based microarray was performed. No significant changes were observed for α-synuclein (SNCA) immunoreactivities regarding CTRL, CTRL IgG, Buffer group and α-synuclein Ab group (Fig. 7a). Fibronectin (FN1) showed a significantly increased immunoreactivity 3 weeks after stable IOP elevation (65726 ± 37398 U) compared with CTRL, CTRL IgG, Buffer groups (15451 ± 14490 U, 4566 ± 1560 U, 19208 ± 10469 U, all p < 0.001, Fig. 7b). Furthermore, immunoreactivities to sigal recognition particle 14 kDa protein (SRP14) were significantly increased in α-synuclein Ab group (6714 ± 1584 U) compared with CTRL (3178 ± 593 U, p < 0.0002), CTRL IgG (4935 ± 1550 U) and Buffer group (4177 ± 905 U, p < 0.0002) 7 weeks after IOP elevation, when animals were sacrificed (Fig. 7c).

### Proteomic analysis and Gene Ontology annotation of IOP elevated eyes

**Figure 8:**
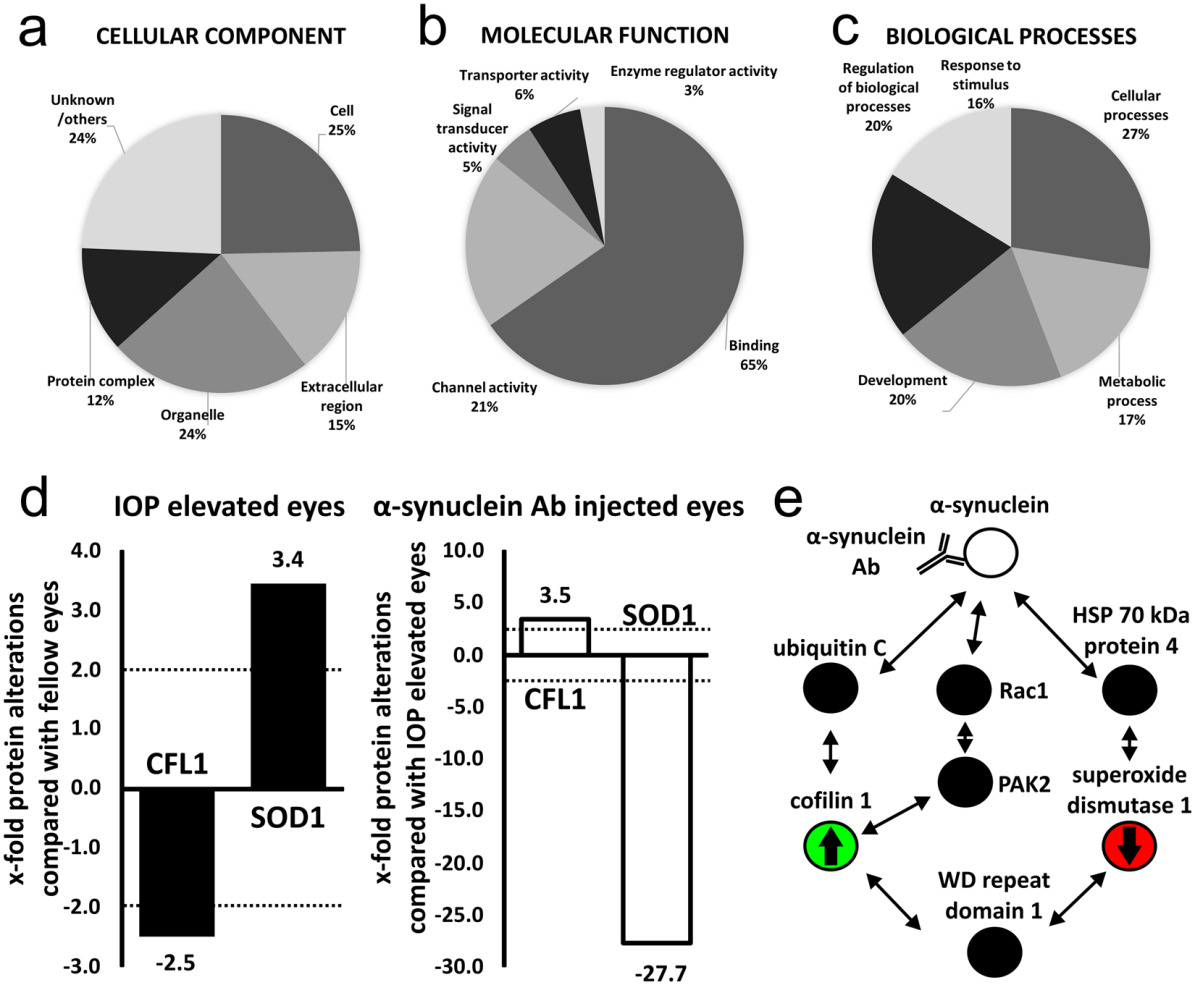
Mass spectrometric analysis revealed neurodegenerative and neuroprotective pathways. Proteins were isolated from retinae and digested to peptides, which were ionized for mass spectrometric analysis. Mass chromatograms provide information for protein identification. Regulations of more than two-fold were regarded as distinct (dotted line) Results from mass spectrometry revealed the proteome of the retina isolated from IOP elevated eyes. GO annotation was performed for (**a**) cellular component, (**b**) molecular function and (**c**) biological processes. (**d**) Retinal protein alterations of retina of IOP elevated eyes were compared with fellow eyes (black bars). The retinal proteome of α-synuclein Ab injected IOP elevated eyes was compared with the retinal proteome of IOP elevated eyes to reveal the neuroprotective mechanisms (white bars). Cofilin 1 (CFL1) is downregulated in the retinal proteome of IOP elevated eyes (−2.5x), but upregulated in the retinal proteome of eyes after α-synuclein Ab injection (3.5x). Superoxide dismutase 1 (SOD1) is upregulated in the retinal proteome of IOP elevated eyes (3.5x) and distinctly downregulated in the retinal proteome of eyes after α-synuclein Ab injection (−27.7x). (**e**) An overview of possible reactions after formation of α-synuclein Ab/protein complex based on STRING protein interaction analysis. Cofilin 1 (green), a major protein involved in the actin cytoskeleton organization, is upregulated while superoxide dismutase 1 (red), a major antioxidant enzyme, is downregulated in the retinal proteome of α -synuclein Ab injected IOP elevated eyes. It is possible that the formation of α -synuclein Ab/protein complex (white) influences downstream signaling pathways, having an effect on the expression level of cofilin 1 and superoxide dismutase 1. It was shown previously that phosphorylation of cofilin 1 via PAK2 and Rac1 is influenced by the presence of α-synuclein protein.

Proteomic analysis followed by Gene Ontology annotation revealed an abundance of proteins with altered protein expression, which were consequently assigned to “cellular component”, “molecular function” and “biological process” categories as designated by the Gene Ontology database. The five categories in cellular component with the greatest proportion of altered proteins were cell (25%), organelle (24%), extracellular region (15%), protein complex (12%) and unknown/others (24%, Fig. 8a). The five categories in molecular function with the greatest proportion of altered proteins were binding (65%), channel activity (21%), transporter activity (6%) and enzyme regulator activity (3%, Fig. 8b). Furthermore, the altered proteins were identified in dependency to the biological processes which were cellular processes (27%), regulation of biological processes (20%), metabolic processes (17%) and response to stimulus (16%, Fig. 8c).

Regarding neuroprotective mechanisms after α-synuclein Ab injection, the two most interesting proteins are CFL1 (3.5x) and SOD1 (−27.7x, Fig. 8d). A complete list of proteins regulated more than two-fold is shown in Supplement 1.

## Discussion

This study allows the following hypotheses regarding the influence of α-synuclein Abs in a rat model of episcleral vein occlusion:

(i) Long-term moderate IOP elevation leads to a neuronal loss of roughly 30% in retinal ganglion cells and axons in CTRL, CTRL IgG and Buffer group.
(ii) Intravitreal injection of α-synuclein Abs appears to have neuroprotective properties especially in the central region of the retina in an experimental animal model of glaucoma.
(iii) α-synuclein Abs reach the GCL, IPL and INL of the retina and seem to be incorporated by RGCs.
(iv) Neuroprotective pathways triggered by α-synuclein Abs might enhance actin cytoskeleton organization and regulate apoptotic processes negatively.

In the current study, the ability of α-synuclein Abs to decelerate neurodegenerative processes in a glaucoma animal model was tested. In recent years, the usage of immunotherapy targeting effector proteins to modulate pathogenic downstream pathways of a vast variety of immune component-related diseases increased more and more. Compared with fellow eyes, IOP was stably elevated 1.5 to 1.9 fold using episcleral vein occlusion^30^, a method that was introduced in 1995 by Shareef *et al*.^31^. Using this method, three episcleral veins are occluded by heat to block the venous outflow, consequently. Since no arteries are occluded in this model, ischemic conditions are rare and seldomly described for this model of ocular hypertension. The slow progressive loss of around 30% RGCs after IOP elevation suits the slow progressive characteristics of glaucoma^32^. A total of 19% (n = 4) did not show stable IOP elevation, which is in line with other publications^33, 34^. Those animals were excluded from the study.

Neurodegenerative processes were observed in IOP elevated eyes of CTRL, CTRL IgG and Buffer group and compared with fellow eyes by a decrease of −11.2%, −10.5% and −13.3% in RNFLT, respectively. Furthermore, an increase of axon damage grade accounted for +83.3%, +80.1% and +97.7%, respectively. To confirm the neurodegeneration in the optic nerve, the density of RGCs was performed. A loss of −31.9%, −30.9% and −29.5% was observed for CTRL, CTRL IgG and Buffer group. This is in line with a loss of −29.5% in previous glaucoma animal models applying episcleral vein occlusion, where RGCs were stained retrogradly using 5% FluoroGold injection in the superior colliculus 8 weeks after IOP elevation^30^. Moreover, the distribution of RGC loss was not uniform. RGCs in the peripher retina were more vulnerable to IOP elevation with a decreasing susceptibility of RGCs towards the optic nerve head^35^.

To further investigate the molecular mechanisms underlying the observed neurodegeneration, Gene Ontology annotation of the retinal proteome of IOP elevated eyes was performed. Many proteins with binding and channel activity function were identified. This implicates the modulation of channel activities for ions, potentially leading to an increased calcium influx and chloride efflux stimulating the apoptotic cascade, finally leading to the decay of RGCs^36^. However, mass spectrometric analysis of the retina does not reveal protein expression changes in specific layers of the retina or cell types, therefore leaving the source of these protein changes obscure.

The potential neuroprotective role of α-synuclein Abs was examined in this study. After an intravitreal injection of 25 µg of a commercially available Ab at the timepoint of stable IOP elevation, a reduction of neurodegenerative processes could be observed. The loss of RNFLT only accounted for 3.9%, axon damage was increased by only 43.0% and RGC density was reduced by 18.1% compared with fellow eyes. For RNFLT and RGC density analysis, the α-synuclein Ab group showed statistically significantly higher levels than CTRL, CTRL IgG and Buffer groups. Furthermore, the α-synuclein Ab group showed highest RGC survival in the central region of the retina with a loss of only 14.7% compared with fellow eyes.

The destination of the previously injected α-synuclein Ab was investigated in histological slices of the retina and distinct fluorescent signals were mainly observed in the GCL, and to a minor extent, in IPL and INL of α-synuclein Ab injected eyes. A colocalization of α-synuclein Ab deposits and Brn3a-positive cells could be shown, probably indicating an uptake of α-synuclein Abs into RGCs and manipulating the intrinsic growth control pathways of RGC axons and possibly processing alterations on signal cascades downstream. Overall, α-synuclein Ab deposits could be detected even seven weeks after injection, indicating a rather long lifetime of the antibody and a fast intracellular uptake. As a control, the intracellular uptake of Control IgGs in the respective group was monitored and showed a 1.5x higher presence of IgG deposits than fellow eyes.

The overall intensity of α-synuclein protein in the retina did not change in the experimental groups. However, one needs to keep in mind that the relative intensity of immunhistological stainings do not give information about the functionality of proteins, but only about their presence, which is presumably the case in α-synuclein Ab group after formation of α-synuclein protein/Ab complex.

Previous studies have shown that serum immunoreactivities vary greatly in intensity in glaucoma and glaucoma animal models^8, 19, 37^. To identify if α-synuclein Ab injection in combination with IOP elevation have an influence on humoral immunoreactivities, an antigen-based microarray was performed. Immunoreactivities against α-synuclein did not change in investigated groups over time. This could indicate that levels of antibodies remain nearly stable during this glaucoma animal model and that injections of exogenous α-synuclein Abs might have no effect on the overall concentration of endogenous α-synuclein Abs. Interestingly, immunoreactivities against fibronectin were significantly increased three weeks after stable IOP elevation. Increased levels of fibronectin Abs could indicate increased expression of fibronectin proteins. Fibronectin is a multimodular glycoprotein that regulates cell adhesion, migration, wound healing beyond other cellular and developmental functions^38^. A direct transmembrane connection between extracellular fibronectin and intracellular actin bundels has been suggested earlier^39^. This is a first hint that modulations of the cytoskeletal organisation and expression alterations of its main protein components could play a significant role in the increased neuronal survival found after α-synuclein Ab injection. Furthermore, signal recognition particle 14 (SRP14) was found to be significantly upregulated in α-synuclein Ab group compared with CTRL and Buffer group 7 weeks after stable IOP elevation. SRPs are highly conserved proteins which catalyze targeting of nascent secretory and membrane proteins to the protein translocation apparatus^40^. Interestingly, upregulated intensities of SRP14 were found in the protein export pathway in Thyroid-associated ophthalmopathy, a disease which represents the first extrathyroidal manifestation of Graves’ disease, a chronic autoimmune disorder affecting the orbit around the eye. The increased expression of SRP14 might be in correlation with functional abnormity of protein export^41^.

Mass spectrometric analysis revealed a number of proteins, which were differently expressed in retina after IOP elevation alone and after IOP elevation followed by α-synuclein Ab injection. Cofilin 1 and superoxide dismutase 1 were of major interest due to their interaction with α-synuclein protein, which was identified with STRING protein interaction analysis (Fig. 8e).

Cofilin 1 (CFL1) has been downregulated in the retinal proteome of IOP elevated eyes and increased in α-synuclein Ab injected animals. It is an ubiquitous actin-modulating protein that catalyzes the rapid assembly and disassembly of actin in interaction with ADP2/3, thus regulating the cytoskeleton dynamics. Moreover, it is involved in endocytosis, angiogenesis and apoptosis^42^. Under stress conditions, cofilin 1 is activated by phosphatases^43^ and forms rod-shaped cofilin-saturated actin filament bundels, a process called cofilin-actin rod stress response^44^. Comparable to our results after elevated IOP stress, downregulated protein levels of cofilin 1 have also been described after stimulation of microglia using nitrated α-synuclein in a Parkinson’s disease model^45^. *In vitro* studies have shown that the presence of α-synuclein leads to the activation of Rac1, a protein known to promote actin polymerization, which results in increased phosphorylation of PAK2 and finally leading to the phosphorylation of cofilin 1 (Fig. 8e)^46^. Inactivation of α-synuclein by α-synuclein Abs could therefore lead to actin stabilization and higher resistance to depolymerization. An upregulation of cofilin 1 in the retinal proteome of α-synuclein Ab group could indicate an improved cytoskeletal organization. Furthermore, cofilin 1 has been shown to negatively regulate apoptotic processes^47^, indicating that elevated protein levels of cofilin 1 might trigger increased neuronal survival.

Superoxide dismutase 1 (SOD1), an antioxidant key enzyme that catalyzes the convertion of cell-damaging superoxide into oxygen or hydrogen peroxide, belongs to the antioxidant defense strategies of the cell and uses copper and zinc as cofactors^48^. It is known as a positive regulator of apoptotic processes^49^. In rodent retina, superoxide dismutase 1 is expressed in the inner limiting membrane, nerve fiber layer, GCL and the pigment epithelium^50^. Increased levels of superoxide dismutases were shown in the aqueous humor of not only primary open angle glaucoma, but also primary angle closure glaucoma patients compared with cataract patients, propably as a stress response to elevated levels of superoxide anions^51^. In a model of retinal ischemia, mitochondrial superoxide dismutase expression was significantly upregulated after injury and later downregulated after intraperitoneal injection of Brimonidine, an alpha 2-adrenergic recerpor agonist. The authors suggested that increased superoxide dismutase expression levels might be a compensatory mechanism to protect RGCs against glutamate excitotoxicity-induced oxidative stress after ischemic injury^35^. We observed a dramatic downregulation of superoxide dismutase 1 in the retinal proteome of α-synuclein Ab group in comparison to the retinal proteome of IOP elevated eyes. This downregulation could reflect a compensatory mechanism based on lower levels of oxidative stress.

The STRING protein interaction analysis revealed a network of α-synuclein, ubiquitin C, cofilin 1, WD repeat domain 1, superoxide dismutase 1 and HSP70 kDa protein 4. On one side, α-synuclein, ubiquitin C, superoxide dismutase 1 and HSP70 kDa protein 4 are involved in stress response due to DNA damage, excessive superoxide radicals and unfolded proteins, respectively. On the other side, cofilin 1, Rac1, PAK2 and WD repeat domain 1 are involved in the actin cytoskeleton organization, enabling the cell shape to adapt and to adjust to altered environmental conditions (Fig. 8e).

The neuroprotective effect of α-synuclein Abs has been described before in a mouse model of synucleinopathy, where the Ab improved clearance of extracellular α-synuclein after immunization against α-synuclein or stereotaxic administration into the brains^52^. Lee and colleagues reviewed mechanisms of α-synuclein based immunotherapy, where they suggested two pathways to be involved: (1) antibodies bind to extracellular α-synuclein protein, blocking the direct transfer of this complex into neurons and (2) facilitating the antibody-assisted internalization of extracellular α-synuclein protein upon binding of the α-synuclein Ab to the Fcɣ-receptors of microglia for lysosomal degradation^53^. However, we were not able to show an intracellular uptake of α-synuclein Ab by microglia, which could be due to lysosomal degradation processes, making the detection of those antibodies impossible seven weeks after initial injection. To prove that specifically α-synuclein antibodies and not any kind of antibody have an effect on neuronal survival, we injected CTRL IgGs in a control group, as described above. Beside binding to α-synuclein protein, it is also possible that α-synuclein Ab could show a cross-reactivity to other proteins. Some antibodies have been proven to bind to more than one target epitope. For example, the use of anti-GFAP antibodies in neuroretinal cell culture showed a neuroprotective effect by reducing oxidative stress. Surprisingly, anti-GFAP antibodies showed a cross reaction with endoplasmic reticulum resident protein 57, possibly triggering additional protective effects^11^.

Taken together, α-synuclein Ab demonstrated a therapeutic usefulness as target for antibody-based therapy in this glaucoma animal model. Mass spectrometric data presented in this study showed alterations of some retinal protein expression involved in cellular stress response and others involved in actin cytoskeleton organization, possibly indicating the molecular mechanisms which prevent enhanced neurodegeneration after α-synuclein Ab injection. A more detailed analysis of the specific targets of α-synuclein Ab could help to improve our knowledge about protein-protein interactions, leading to an enhanced insight of the molecular mechanisms of α-synuclein Ab.

## Methods

### Animals and anesthesia

All experiments were conducted in accordance with the Association for Research in Vision and Ophthalmology Statement on the Use of Animals in Ophthalmic and Vision and approved by the national investigation office in Koblenz, Germany (23 177-07/G 15-1-053). Female Sprague-Dawley rats (200 g) were obtained from Charles River (Sulzfeld, Germany) and housed in climate-controlled rooms with 12 h light-dark cycle and fed ad libitum. Anesthesia was induced by intramuscular injection of 0.185 ml/kg body weight medetomidine hydrochloride (1 mg/ml Dorbene vet., Pfizer, New York, NY) and reversed using atipamezol hydrochloride (5 mg/ml Alzane, Zoetis Österreich GmbH, Wien, Austria). Animals were anesthetized for surgery, intravitreal injection and optical coherence tomography (OCT). Episcleral vein occlusion (EVO) by cauterization was performed unilaterally on the left eye, the right fellow eye served as the control.

### Experimental design

All rats underwent the same routine examinations as IOP measurement, OCT analysis, and blood withdrawal. Additionally, all rats underwent surgery for subsequent IOP elevation. Animals without successful IOP elevation were excluded from the study (n = 4). Control group (n = 6) received no injection in the left eye. CTRL IgG group (n = 5) received a total volume of 2.5 µl containing 25 µg CTRL IgG as intravitreal injection of commercially available rabbit-derived Control IgG without a known specificity (Abcam, Cambridge, UK). Buffer group (n = 6) received a buffer injection (2.5 µl, Tris 0.1 M, glycine 0.1 M) in the left eye. The α-synuclein Ab group (n = 5) received the injection of rabbit-derived α-synuclein Abs in buffer (2.5 µl, Tris 0.1 M, glycine 0.1 M) once after IOP elevation was stable. Seven weeks after intravitreal injection, all animals were humanely sacrificed by carbon dioxide exposure. Methods are described in detail in the subsections.

### IOP measurement

A TonoLab rodent rebound tonometer (icare, Espoo, Finland) enables accurate IOP measurements in a non-invasive manner. Care was taken to hold the unsedated animal in a horizontal position on an arm with the head fixed very gently in a neutral position with a hand without applying pressure to the animal. The tonometer was kept in a vertical position and ten consecutive measurements were calculated as one mean value. Instrument-generated outlier or outlier due to movement of the animal were excluded from not recorded. For comparability, IOP measurements were performed between 9 am and 12 pm every week on both eyes.

### Experimental glaucoma development

**Figure 9:**
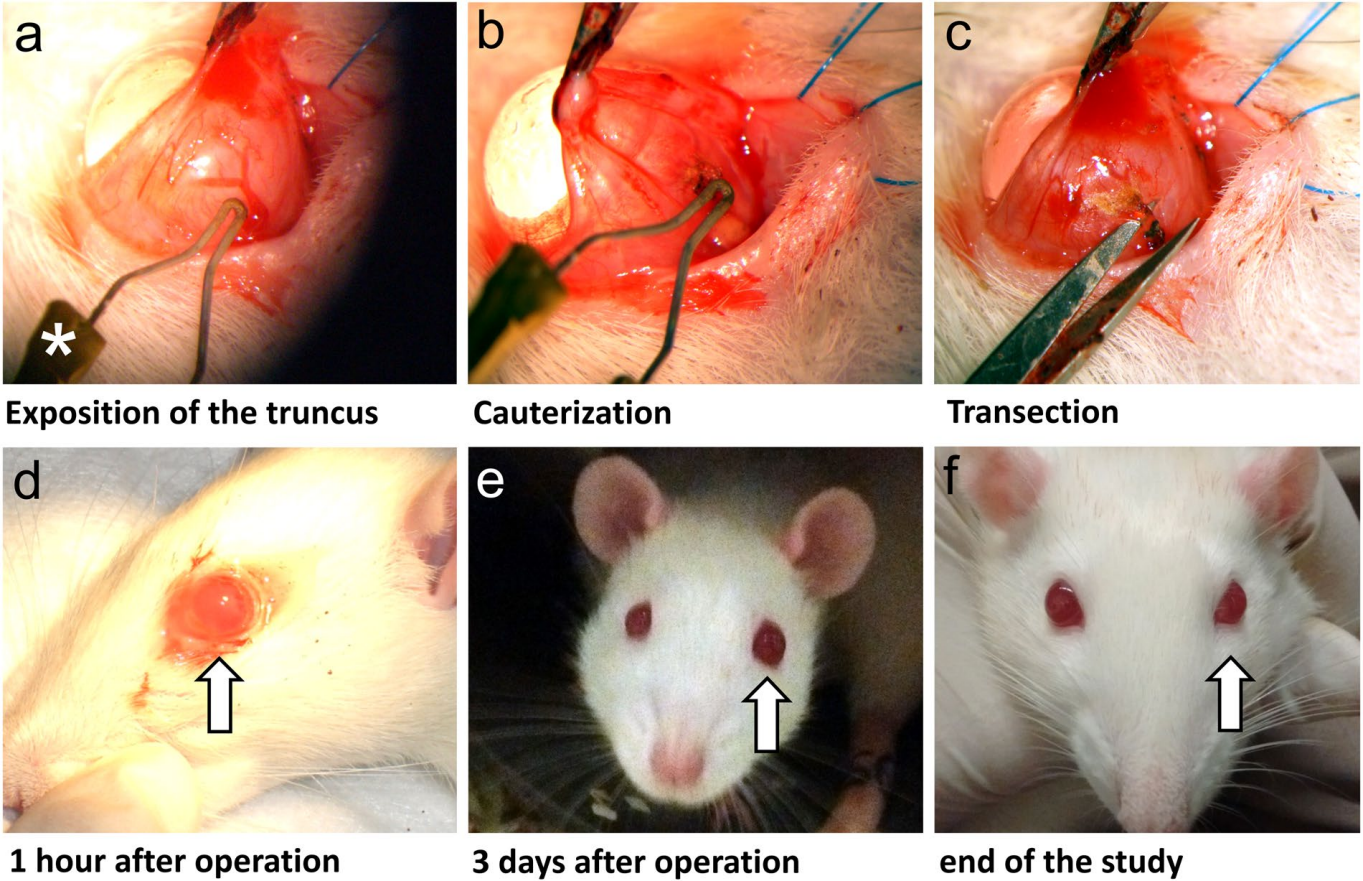
The occlusion of episcleral veins by cauterization and full recovery of the eye after operation. Representative figures of the cauterization of episcleral veins show the exposition of the truncus (**a**) after incision of the conjunctiva and the cauter (*). The venous truncus was cauterized with great attention not to damage surrounding tissue (**b**) and remaining tissue was transected using a scissor (**c**). Recovery from the operation occurred rather fast as shown after 1 hour (**d**), 3 days (**e**) and at the end of the study (**f**).

The IOP of one eye per animal was elevated using the model of episcleral vein occlusion^31^. Therefore, the conjunctiva was incised and three major venous trunks in nasal, superior and inferior position were exposed. Ophthalmic cautery followed by a transection of the remaining tissue led to a consequent partial venous outflow, resulting in elevated IOP levels (Fig. 9a–c). The conjunctiva was readjusted and animals received three drops of Novaminsulfon (500 mg/ml Metamizole, ratiopharm GmbH, Ulm, Germany) diluted in water for pain control. After surgery, animals recovered quickly (Fig. 9d–f) and were observed daily for pathological changes such as inflammation or edematous cornea. Ischemic conditions were not observed. Animals without IOP elevation after EVO were excluded from the study (n = 4).

### Intravitreal injections of α-synuclein antibodies

Intravitreal injection in the left eye was performed once after IOP levels achieved stable elevated levels (3 weeks after EVO). A total volume of 2.5 µl containing 25 µg α-synuclein Ab (SNCA, rabbit polyclonal, ABIN1031834, antibodies-online.com) was injected intravitreally using a 30 G needle and a 10 µl glass Hamilton syringe (Sigma Aldrich, Steinheim, Germany). Dosage and volume are comparable to a study highlighting the effect of intravitreally injected ranibizumab, a monoclonal antibody, in a rodent model of diabetes^54^. Great care was taken not to damage the lens or disturb the retinal blood supply. The needle was kept intravitreally for 15 seconds to avoid major efflux. Eyes of the Buffer group were injected with 2.5 µl buffer solution equal to the α-synuclein Ab buffer. Fellow and control eyes received no intravitreal injection. The animals eyes were monitored weekly to detect inflammatory alterations such as vitreous haze or other events such as reogranization of retinal layers or edema using OCT. *Ex vivo*, retinal cross sections were examined for infiltrating cells or other acute pathological changes. No changes were observed for eyes injected with α-synuclein Ab.

### Monitoring the retinal nerve fiber layer using optical coherence tomography

Changes of the retinal nerve fiber layer thickness (RNFLT) were accessed *in vivo* using SD-OCT (Spectralis OCT + HRT, Heidelberg Engineering GmbH, Heidelberg, Germany). Pupils of anesthetized rats were dilated by topical administration of tropicamide (Mydriaticum, Pharma Stulln, Stulln, Germany) and eyes were kept moist with hyaluronic acid (Artelac Splash, Bausch + Lomb, Rochester, NY, USA). To improve the image quality, a contact lens (Cantor + Nissel, Northamptonshire, England, PMMA 2.70/5.20, radius of curvature of the central optic zone: 2.70 mm; diameter: 5.20 mm) was placed onto the cornea after topical application of 0.5% proparacaine (URSA-Pharm, Saarbrücken, Germany) for local anesthesia of the cornea. Animals were placed in a neutral position on a custom-built imaging stage. Several rodent-specific adaptations needed to be performed for proper OCT acquisition: The corneal radius was set to 7.7, the reference arm and focus was adjusted manually for each eye for maximal clarity and quality. The real-time eye tracking function was used to reduce noise by averaging 100 images to one overlay image and the follow-up function was used to ensure the identical location during all longitudinal measurements. For each eye, one 12° diameter circular B-scan with 100 frames was taken and analyzed. Analysis was performed with the Heidelberg Eye Explorer software (version 1.9.10.0). The segmentation algorithm is designed to recognize human retina, therefore, it was necessary to edit manually and individually the RNFL segmentation line for each scan (Fig. 2a–c). OCT examination was performed with all animals three weeks after EVO and eight weeks after intravitreal injection.

### Quantification of axonal loss in optic nerves

Optic nerve neurodegeneration was accessed after p-phenylenediamine (PPD) staining of 0.7 µm semithin transverse optic nerve sections as described previously^55^. Briefly, six to seven images per optic nerve were captured in a crosswise procedure using 100x magnification (Olympus SPlan 100x/1.25 oil, 160/0.17, Olympus Deutschland GmbH, Hamburg, Germany) and an upright microscope (Olympus Vanox-T AH-2;) in accordance with a consistent orientation. Every image represents 7800 µm^2^ of the optic nerve. An overall coverage of 36.0 ± 11.8% of the total optic nerve cross section was achieved (Fig. 3a). The damage level of paraphenylendiamine-stained optic nerve cross sections was analyzed according to a previously introduced grading scheme^56^. Representative images are shown for each group (Fig. 3b–d). Analysis was performed by two investigators in a blinded manner.

### Immunohistochemical analysis of retinal ganglion cells in retinal flatmounts

Immediately after sacrificing the animals with carbon dioxide, eyes were enucleated, transferred to 8 °C PBS and incised circumferentially. The retina and sclera were incised four times to produce a cloverleaf-like shape. Retina was carefully separated from the sclera, mounted on a filter and fixed in 4% paraformaldehyde (pH 7.4) for 30 minutes. After two wash steps with PBS, the retina was kept in 30% saccharose/PBS (pH 7.4) for 24 hours. Retina was frozen in 2-methylbutane and liquid nitrogen and stored at −80 °C. Retinae were washed in PBS twice, and incubated for 90 minutes in 10% FCS + Triton-X-100. Brain-specific homeobox/POU domain protein 3 A (Brn3a) has proven to be an excellent marker for the whole population of RGC in the adult rat retina^57^. Incubation of the retina using primary Ab goat anti-Brn3a (C20, 1:400 in 10% FCS-PBS, sc-31984, Santa Cruz Biotechnology, Santa Cruz, CA, USA) was done overnight at 4 °C. Residual Ab was removed with PBS for 10 minutes twice. Slides were blocked for 30 minutes in 10% FCS + Triton-X-100 and incubated with secondary Ab donkey anti-goat IgG (H + L) Alexa Flour 568 (1:400 in 10% FCS-PBS, A-11057, Life Technologies, Carlsbad, CA, USA) for two hours. Residual Ab was discarded with PBS and retina was mounted RNFL side up on microscope slides and covered with antifading solution for microscopy (VECTASHIELD Antifade Mounting Medium, H-1000, vector laboratories, Burlingame, CA, USA). Brn3a-positive cells were visualized using an Eclipse TS 100 microscope (Nikon, Yurakucho, Tokyo, Japan) with a DS-Fi1-U2 digital microscope camera (Nikon) and an ELWD 20x/0.45 S Plan Flour Ph1 ADM objective (Nikon) and recorded by the imaging software NIS Elements (Nikon, Version 4.10 64 bit). 10 images, each representing 0.14 mm^2^ of the retinal quarter and assigned a region (central, middle, peripher) depending on the distance to the optic nerve head, were taken. Images covered 20.7 ± 3.7% of a retinal quarter. Retinal ganglion cell density was analyzed manually and semi-automated using ImageJ cell counting software (http://rsb.info.nih.gov/ij/, NIH, Bethesda, MD, USA). The macro included the following steps: (1) convert to 8-bit, (2) subtract background (3) set auto threshold (4) run “nucleus counter” with smallest 800 and largest 7000. Analysis was performed by two investigators in a blinded manner.

### Immunohistochemical analysis of α-synuclein antibodies, IgG deposits and α-synuclein protein in retinal cross sections

After immersion-fixation in 4% formaldehyde (Histofix, Roth, Karlsruhe, Germany), the specimen was embedded in paraffin using a standard histologic technique in a proper orientation to allow the visualization of the retinal architecture and its distinct layers. The specimen block was sectioned at 10 µm intervals with a microtome (LEICA REICHERT JUNG 2030, Leica, Rijswijk, Netherlands). For each staining, six sections per eye were prepared. The sections were flattened in a water bath at 40 °C and mounted onto slides that were stored at 60 °C overnight. Sections were deparaffinized und slowly rehydrated with descending alcohol washes (100%, 100%, 95%, 95%, and 70%) and finally rinsed in Aqua dest. An antigen retrieval in DAKO’s Target retrieval solution (95 °C, 40 minutes; DAKO, Hamburg, Germany) and a blocking step was performed. Deposits of α-synuclein Abs were visualized after incubation with a goat anti-rabbit IgG (H + L) Cy3 (1:1000, ZRV1158, Linaris GmbH, Dossenheim, Germany) in PBS for 1 hour. IgG deposits were visualized using goat anti-rat IgG (H + L) TRITC (1:200, GenWay Biotech Inc., CA, USA) and nuclei were visualized with DAPI (VECTASHIELD Mounting Medium with DAPI, H-1200, vector laboratories, Burlingame, CA, USA). For documentation, an Eclipse TS 100 microscope (Nikon, Yurakucho, Tokyo, Japan) with a DS-Fi1-U2 digital microscope camera (Nikon) and an ELWD 40x/0.45 S Plan Flour Ph1 ADM objective (Nikon) were used. The number of α-synuclein Ab deposits per retinal layer were calculated per mm of the retinal cross section. The number of IgG deposits in the ganglion cell layer were calculated per mm retina.

The expression level of α-synuclein protein in the retina was investigated in retinal cross sections as described before. The rabbit α-synuclein primary Ab (ABIN1031834, antibodies-online.com) was diluted 1:100 in DPBS and incubated on six cross sections per eye overnight at 4 °C. Samples were incubated with a goat anti-rabbit IgG (H + L) Cy3 (1:1000, ZRV1158, Linaris GmbH, Dossenheim, Germany) in PBS for 1 hour and nuclei were visualized with DAPI (VECTASHIELD Mounting Medium with DAPI, H-1200, vector laboratories, Burlingame, CA, USA). The signal intensity of the total retina and the inner layers of the retina was recorded with ImageJ and compared within groups.

### Blood withdrawal

The animal’s tail was immersed in warm water for one minute to dilate blood vessels and dried afterwards with a paper towel. To yield longitudinal data of antibody reactivities, blood was taken at baseline, 3, 5 and 7 weeks after IOP elevation from the lateral tail vein, while the rats were fixed in an animal restrainer. Blood samples clotted at room temperature and were centrifuged at 10 °C for 10 min and 4000 rpm. Serum was eluted carefully and stored at −20 °C.

### Antigen-based microarray

To investigate, whether the IOP elevation has an impact on IgG autoantibody reactivities, an antigen-based microarray analysis was performed. In this microarray approach, serum was used as source for primary Abs. A setup of specific antigens of interest for glaucoma research (Supplementary Table S1) were spotted in triplicates using a non-contact array spotter (sciFLEXARRAYER 3, Scienion, Berlin, Germany) on glass slides (Oncyte, nitrocellulose 16 multi-pad slides, Grace Bio-Labs, Bend, OR, USA). Each glass slide contained 16 nitrocellulose coated areas (subarrays) with the complete spotting pattern. All incubation steps were performed at 4 °C under slight agitation. Unspecific binding sites of the nitrocellulose membrane were blocked by incubation with 100 µl of 0.5% bovine serum albumin (BSA, Sigma Aldrich, Steinheim, Germany) in 0.5% Tween 20 in phosphate buffered saline (PBS-T, Gibco life technologies, Carlsbad, CA, USA) for 1 hour. Slides were washed three times for 10 minutes with PBS-T before they were incubated with 2 µl serum diluted 1:250 in PBS overnight. After three washing step with PBS-T for 10 minutes, the secondary Ab goat anti-rat IgG (H + L) Cy5 (Invitrogen, Carlsbad, CA, USA) was diluted 1:250 in PBS and incubated on membrane for 1 hour. A final washing step with PBS-T once and ultra-pure water (HPLC-H_2_O, AppliChem, Darmstadt, Germany) twice for 15 minutes followed. Slides were dried afterwards (SpeedVac Concentrator 5301; Eppendorf, Hamburg, Germany). Emitted fluorescence signals were scanned with the high-resolution confocal array scanner (Affymetrix 428, Santa Clara, CA, USA) at 10 dB. Digitized signals were analyzed with Imagene 5.5 (BioDiscovery Inc., Los Angeles, CA, USA).

### Mass spectrometric analysis of the retinal proteome

Retinae were frozen in 2-methylbutane (VWR Chemicals, Radnor, PA, USA) and liquid nitrogen and stored at −80 °C until mass spectrometric analysis. Liquid nitrogen snap frozen retina was grinded using a pestle until a powder emerged, which was resolved in 150 µl lysis buffer (20 mM Tris, 150 mM NaCl, 0.5% DDM in TBS). 1 µl protease inhibitor cocktail (P 1860, Sigma Aldrich, Steinheim, Germany) was added and incubated for 1 h at 4 °C before centrifugation at 14,000 g for 20 min at 4 °C. The supernatant was collected for total protein measurement using the PierceTM BCA Protein Assay Kit (Thermo Scientific, Rockford, IL, USA) following the manufacturer’s protocol. 20 µg of total protein per sample was loaded on individual wells on a 12% Bis-Tris 10-well prepared SDS PAGE minigels (Thermo Scientific, Rockford, IL, USA) under reducing condition at 150 V followed by Coomassie Brilliant Blue staining (Invitrogen, Carlsbad, CA, USA). Individual lanes were cut corresponding to four mass migration areas following trypsin based in-gel digestion, following a modified protocol^58^. Thereby, destained and dehydrated gel pieces were incubated with 60 µl sequencing grade modified trypsin (10 µg/µl, Promega Corporation, Madison, WI, USA) overnight at 37 °C following peptide extraction and vacuum lyophilization in a SpeedVac. Lyophilized peptides were purified by ZIPTIP® C18 solid phase extraction (Millipore, Billerica, MA, USA) following the manufacturer’s protocol. Resolubilized peptides (20 µl 0.1% TFA/piece digest) were analyzed by use of an established LC ESI MS system implementing an LTQ Orbitrap XL instrument (Thermo Fisher Scientific, Rockford, IL, USA)^59^. Raw files were combined and matched against the SwissProt human database (SwissProt 150301) using Mascot (version 2.2.07) and Proteome Discoverer (version 1.1, Thermo Fisher Scientific, Rockford, IL, USA). Carbamidomethylation was set as a fixed modification. The tolerance in mass precision for MS/MS was 20 ppm and 0.5 Da. All identified proteins were annotated according to the Gene Ontology database (http://www.geneontology.org) using Cytoscape version 2.8.3 with integrated BINGO 2.44 plugin (www.cytoscape.org), and ontology categories for Cellular Component, Molecular Function, and Biological Processes. Raw files were transferred to Excel. Fold changes two-fold or higher were regarded as distinct regulations of proteins. The retinal proteome of IOP elevated eyes was compared with fellow eyes, while the retinal proteome of α-synuclein Ab injected eyes was compared with the retinal proteome of IOP elevated eyes to analyze neuroprotective processes. Strong protein protein interactions were analyzed using STRING version 10 with the following settings: highest confidence (0.900), no more than 20 interactors^60^ (http://string-db.org).

### Data procession and statistical analysis

Data are given as mean ± standard deviation (SD). Statistical analysis was performed using Statistica Version 12 (Dell Inc. Round Rock, TX, USA). Differences among multiple groups were analyzed by one-way analysis of variance, followed by Tukey’s HSD post-Hoc (unequal N) tests. Differences between two individual groups were analysed with student’s independent t test by groups. P < 0.05 was considered to be statistically significant. Images were processed with CorelDRAW X4 Graphic Suites (Corel Corporation, Ottawa, Canada) and saved as TIFF. All data generated or analysed during this study are included in this published article (and its Supplementary Information files).

### Ethical approval

All experimental protocols were approved by the national investigation office in Koblenz, Germany (23 177-07/G 15-1-053). The methods were carried out in accordance with the relevant guidelines and regulations.

## Electronic supplementary material

Supplementary Dataset
